# Toward a common clinical lexicon of consciousness

**DOI:** 10.1007/s11739-023-03359-9

**Published:** 2023-07-08

**Authors:** Stephen Bacchi, Sheryn Tan, Mark Slee

**Affiliations:** 1 Department of Neurology, Flinders Medical Centre, SA Health, Flinders Drive, Bedford Park, SA 5042 Australia; 2 College of Medicine and Public Health, Flinders University of South, Adelaide, Australia; 3 University of Adelaide, Adelaide, SA 5000 Australia

**Keywords:** Unconsciousness, Reduced level of consciousness, Unawareness, Coma, Obtundation

Clear, consistent, and universally understood communication is essential to safe and effective medical practice. The use of language for which all parties have a common definition and understanding is a necessary component of this communication. Terminology used in the assessment and management of disturbances in consciousness can at times be variable and imprecise. For example, terms including ‘obtunded’ and ‘stupor’ may have different meanings for individual clinicians. While the neurobiological substrate required for the generation of consciousness is incompletely understood, it is crucial that clinical evaluations of disturbances of consciousness are accurate and reproducible [1]. While there is increasing utility of investigations such as electroencephalogram and functional magnetic resonance imaging (MRI) in the evaluation of consciousness [2], clinical evaluation remains fundamental. Since new disturbances of consciousness can herald a number of life-threatening conditions, clear communication regarding clinical examination findings in these settings is vital.

Definitions of consciousness itself, particularly in basic scientific research, vary significantly. However, clinically, it is generally agreed that for normal consciousness to be present, there are two key components of consciousness that must both be intact: the *level of consciousness* and *content of consciousness*. These components of consciousness are referred to using multiple other terms in the literature, most often wakefulness and awareness respectively (see Supplementary Information 1). These two key components of consciousness are used in society guideline statements and reviews [3]. Terms that are used in the discussion of consciousness, but are not necessarily required for consciousness, may relate to sensory and motor function—namely, *connectedness* and *responsiveness* (see Supplementary Information 2) [4].

There are multiple terms that clinicians may use to describe disturbances of consciousness that have poorly defined or have variable definitions. Such terms include ‘stupor’ and ‘moribund’. Even terms such as ‘drowsy’ may be prone to variable interpretation, including when modifier terms such as ‘pathological drowsiness’ are used. The use of such terms has the potential to lead to misunderstandings, and may limit the reproducibility of assessments when performed by different clinicians. Descriptions of acute or chronic disturbances of consciousness should provide clinical evaluations that are interpretable by subsequent clinicians, focussing on the objective assessment of level of consciousness and content of consciousness (see Table 1). Additionally, terms regarding distinct disorders that may manifest as disturbances of consciousness should be used precisely. For example, ‘delirium’ or ‘delirious’ may be used to refer to a diagnosis, which can cause alterations in arousal and awareness [5]. Therefore, it can be seen that the term ‘delirium’ relates to the *cause* of a disturbance of consciousness (as could the terms such as ‘stroke’ or ‘seizure’), rather than a *description* of the disturbance of consciousness itself.

**Table 1:** Suggestions regarding terms used in the description of acute and chronic disturbances of consciousness

Term	Recommendation	Accepted meaning
Obtundation	Not recommended	Variable use
Stupor	Not recommended	Variable use
Drowsy	Not recommended	Variable use
Lethargy	Not recommended	Variable use
Moribund	Not recommended	Variable use
Somnolent	Not recommended	Variable use
Clouding of consciousness	Not recommended	Variable use
Unarousable (reduced level of consciousness)	Recommended, with specifiers (e.g., unarousable to voice, or unarousable to noxious stimuli). If patient does arouse, then describe (e.g., opens eyes to voice)	Altered state involving impaired wakefulness (i.e., impaired arousal). No eye opening (or voluntary motor activity) following specific environmental stimulus
Unresponsive	Recommended, with specifier (e.g., unresponsive to voice, or unresponsive to noxious stimuli). If patient does respond, then describe (e.g., eyes tracking to visual target)	No behavioural change following specific environmental stimulus
Coma	Recommended	Unarousable unresponsiveness*
Unaware (reduced content of consciousness)	Recommended, in specific circumstances	No perception of environmental or internal subjective experience**

*Noting the caveat that unresponsiveness may, but does not always, indicate unawareness

**Noting the difficulties in evaluating the state of awareness at the bedside, particularly in the setting of disturbances of level of consciousness, currently this term may be best used in the setting of the specific evaluation of chronic disorders of consciousness (such as persistent vegetative state and minimally conscious states)

The evaluation and description of disturbances in level and content of consciousness may employ a variety of strategies, and should take into consideration potential mimics. When evaluating the *level* of consciousness (see Supplementary Information 3), there are multiple validated tools, including the Glasgow Coma Scale (GCS), Full Outline of UnResponsiveness (FOUR) scoring system, and AVPU score. It is now recommended that the FOUR score be used to evaluate level of consciousness in the intensive care unit, rather than GCS [2]. However, each of these scores has limitations of which clinicians should be aware. When evaluating *content* of consciousness (see Supplementary Information 4), specific, consistent descriptions of patient responses to given environmental stimuli should be provided to enable single-point and longitudinal assessment. Investigations such as electroencephalogram and functional MRI may have an increasing role in the evaluation of these components of consciousness [2]. Clinical examples of disorders of consciousness may result in impairment of either component of consciousness, or both (see Supplementary Information 5). Clinicians should be aware of motor and sensory abnormalities that may interfere with the evaluation of consciousness. Identifying mimics for disturbances of consciousness is vital. Typically, these mimics manifest as unresponsiveness, which highlights the importance of acknowledging that unresponsiveness is not inherently synonymous with a reduced content of consciousness. These mimics may include a lack of power precluding motor responses (such as in locked-in syndrome), an inability to initiate motor responses (such as due to akinetic mutism, catatonia, or functional unresponsiveness), and an inability to perceive or understand sensory stimuli (such as due to global aphasia, language barrier, and hearing impairment). The phenomenon of covert awareness, otherwise referred to as cognitive motor dissociation, describes a state in which no outward signs of consciousness are demonstrated but there is imaging or neurophysiological evidence of consciousness and is also relevant to consider in this setting, along with the ethical issues this entity raises (see Supplementary Information 6) [6].

Pragmatically, in a clinical setting, one of the most important aspects of one’s description of disturbances of consciousness is that they may be understood by a second clinician, and re-evaluated such that changes in clinical state (either improvement or deterioration) can be detected. To this purpose, using descriptive terms and describing physical examination findings may be the most useful strategy. For example, when describing level of consciousness, outlining whether a patient’s eyes are open spontaneously, in response to voice or in response to noxious stimuli, may enable repeated evaluation and clear communication. This example highlights the utility of commonly understood grading systems such as GCS. However, while such scales are useful, they are not necessarily sufficient, particularly in the setting of other neurological pathologies that may limit evaluation through pathways other than that of impairments of level or content of consciousness (such as dysphasia). Similar concepts apply when describing responsiveness and awareness. Clear descriptions of the stimuli provided and the response obtained, its reproducibility, and the environmental settings in which the behaviour was observed may be required for a complete description. For example, describing reproducible smiling in response to family on separate occasions conveys significantly different meaning to having observed smiling once without specific environmental stimulus.

Careful evaluation and communication are required in the clinical assessment of disturbances of consciousness. This evaluation involves examination of both the level of consciousness and the content of consciousness. When communicating regarding patients with disturbances of consciousness, ambiguous terms with variable meanings should be avoided. Commonly used scores, such as the GCS, may be useful in specific circumstances, but are not always sufficient. Harmonisation of communication will facilitate longitudinal assessments and will aid in the standardisation of the evaluation of these unwell patients.

## Supplementary Information

Below is the link to the electronic supplementary material.

Supplementary file1 (DOCX 386 KB)
