# Supplementary material for: Toward a common clinical lexicon of consciousness
Source: Intern Emerg Med. 2023 Jul 8;18(6):1851–3. doi: 10.1007/s11739-023-03359-9 (PMC10504159; doi:10.1007/s11739-023-03359-9)
Supplement: Supplementary file 1 — Supplementary file1 (DOCX 386 KB) [file 11739_2023_3359_MOESM1_ESM.docx]

**Supplementary Information 1 –** Terminology used to refer to level of consciousness and content of consciousness

| **Level of consciousness synonyms** | **Content of consciousness synonymous** |
| --- | --- |
| Wakefulness | Appropriate *Awareness* of self (internal) and environment (external) |
| Arousal |  |
| Vigilance |  |
| Alertness |  |
| Sensorium |  |

**Supplementary Information 2 –** Connectedness and responsiveness in relation to consciousness

In the discussion of consciousness, the term connectedness describes the ability of an individual to perceive sensory stimuli from their environment. This concept is useful in the distinction between awareness that relates to the external environment (in which perception of external sensory stimuli is required) and awareness that relates only to the self (in which the perception of external sensory stimuli is not necessarily required) [1]. On the other hand, the term *responsiveness* is used to described behavioural change following environmental stimulus. In a broad sense, responsiveness may be considered to include involuntary responsiveness (including reflex-mediated responses) and voluntary responsiveness. In the evaluation of consciousness, voluntary responsiveness is of the most utility (whereas aspects of reflex involuntary unresponsiveness may be salient in the evaluation of brain death). Since voluntary responsiveness typically manifests through movement or verbal output, it is inherently closely linked with motor function. Therefore, it can be seen that voluntary responsiveness relies not only upon level of consciousness and content of consciousness, but also sensory function and motor function.

Theoretical hierarchy of dependencies*


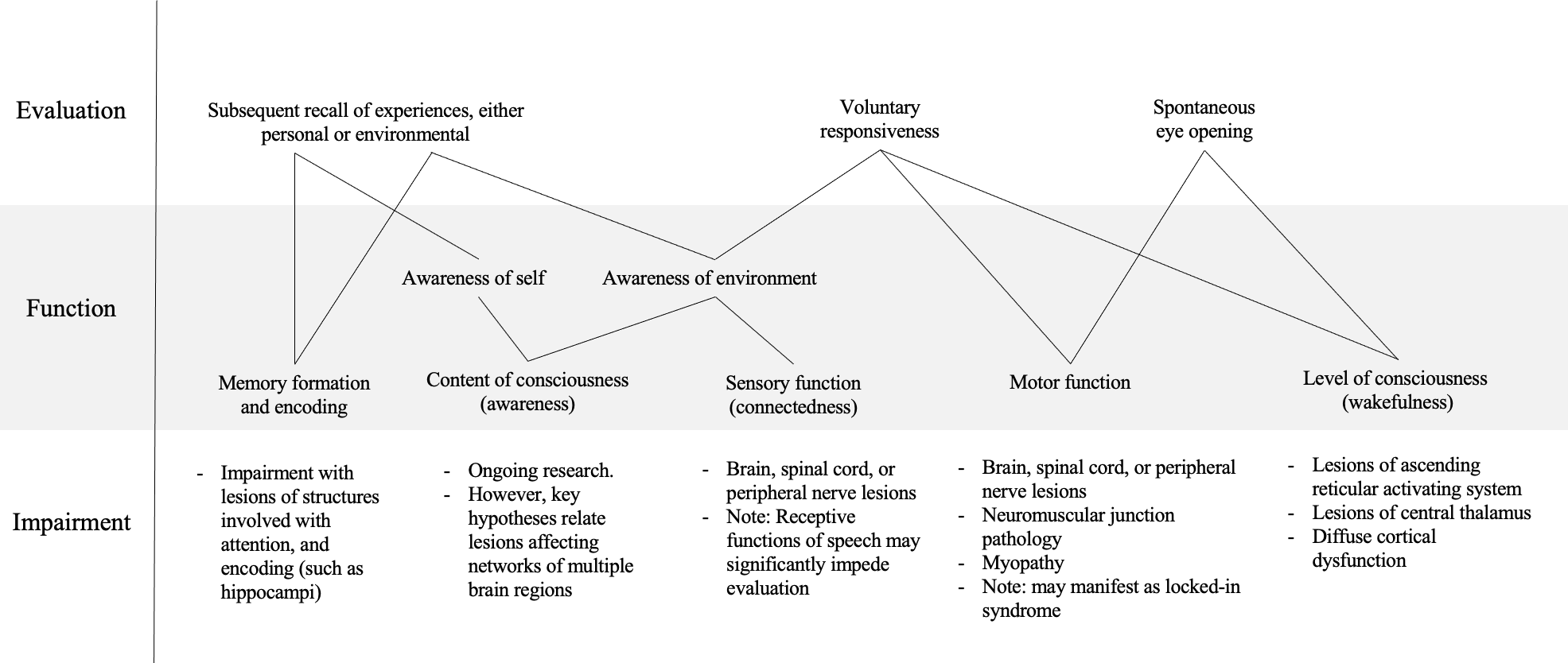


*A deficit in clinical features able to be evaluated (top row), may result from an interruption to any functions associated with that means of evaluation.

**Supplementary Information 3 –** Level of consciousness definition and clinical evaluation

Definition

Level of consciousness may be otherwise referred to as ‘wakefulness’, ‘arousal’, or ‘vigilance’ [2]. Although the term may be less commonly used, ‘alertness’ may refer to the level of consciousness [3]. Wakefulness may be contrasted with sleep, and is generally accepted to be signified by periods of spontaneous eye opening, often with motor activity [4]. However, an intact level of consciousness may also be present with voluntary eye closure, or neuromuscular weakness resulting in eye closure, during which other voluntary responsiveness is demonstrated.

Evaluation

Physical examination to evaluate level of consciousness begins with general inspection. Initially, without stimulation, spontaneous eye opening (or mechanical/neuromuscular factors that may preclude spontaneous eye opening), or other voluntary movements, may demonstrate a relatively unimpaired level of consciousness. When the eyes are not open and there is a lack of voluntary movement, these findings may indicate an impairment in the level of consciousness.

When there is an impaired level of consciousness there are a variety of scales that have been developed to aid with the standardised communication of various degrees of this impairment. These scales include the Glasgow Coma Scale (GCS) [5], the AVPU score [6], the arousal scale in the Coma Recovery Scale-Revised [7], the coma/near-coma scale [8], and the Full Outline of UnResponsiveness (FOUR) scoring system [9]. These scores may each have advantages and disadvantages, and have undergone comparative analyses in a variety of settings [10-12]. For example, GCS has previously been shown to have high inter-observer variability [13], and to frequently be inaccurate when evaluated by junior doctors [14] and emergency care providers [15]. It should be noted that multiple components of these scores rely upon intact motor function (e.g., muscles involved in eye opening and phonation), and may therefore be impaired in the setting of peripheral or central nervous system pathologies that affect these functions.

**Supplementary Information 4 –** Content of consciousness definition and clinical evaluation

Definition

The content of consciousness may otherwise be described as ‘awareness’. This awareness relates to the perception of a subjective experience [16]. As outlined above, external awareness relates to awareness of the surrounding environment. Conversely, internal awareness relates only to oneself.

Evaluation

The evaluation of awareness (content of consciousness) may be significantly more difficult than evaluating wakefulness (level of consciousness). In the setting of a relatively intact wakefulness, clinical methods for the evaluation of awareness typically rely upon the assessment of responsiveness. Examples of responsiveness, in addition to obeying commands, include eye tracking, and smiling or crying in response to family members (as opposed to non-specific smiling or crying). Signs of responsiveness may require close examination over prolonged periods, as seemingly random events may at times indicate awareness when they occur in a repetitive meaningful fashion in response to environmental changes (e.g., a finger movement or eye blink that occurs reproducibly may indicate awareness, and indeed at times may convey attempts at communication). Conversely relatively few observations of unintelligible vocalisation in response to command may be required to demonstrate awareness [17]. Other examples of clinical signs that support the presence of awareness include reaching for objects in a goal-directed manner, and holding and moving objects in a manner that indicates perception of their size, shape, and function (e.g., bringing a comb to one’s hair). However, it should be noted that apparent goal-directed behaviour may occur in settings of impaired awareness (such as frontal lobe seizures), which highlights the requirement for serial assessments in the evaluation of awareness. Further research is ongoing regarding additional physical examination signs that may support the presence of awareness, and incorporate these signs into guidelines [18]. As has been discussed previously, the bedside evaluation of voluntary responsiveness also requires a degree of intact sensory afferents and motor efferents. Accordingly, these techniques may be influenced by multiple factors in addition to the content of consciousness, such as the level of consciousness, motor, and sensory function (see mimics section). Therefore, in the setting of markedly reduced wakefulness, it may be infeasible to clinically assess awareness. It is also noteworthy that awareness may change over time, and this potential for change further highlights the necessity of longitudinal evaluation.

In retrospect, it may be possible to determine that an individual was aware during a specified period of time on the basis of the recall of events from that period, even if they were not responsive at the time [1]. However, lack of recall is consistent with, but not specific to, a period of unawareness. There are many distinct pathologies that may impair attention or memory, some of which may overlap with aetiologies that cause disturbances of consciousness [19].

**Supplementary Information 5 –** Clinical examples of disturbances of consciousness

The previously presented components of consciousness exist on a spectrum. However, to facilitate conceptualisation, these spectra may be dichotomised based upon whether a clinically significant reduction is present.


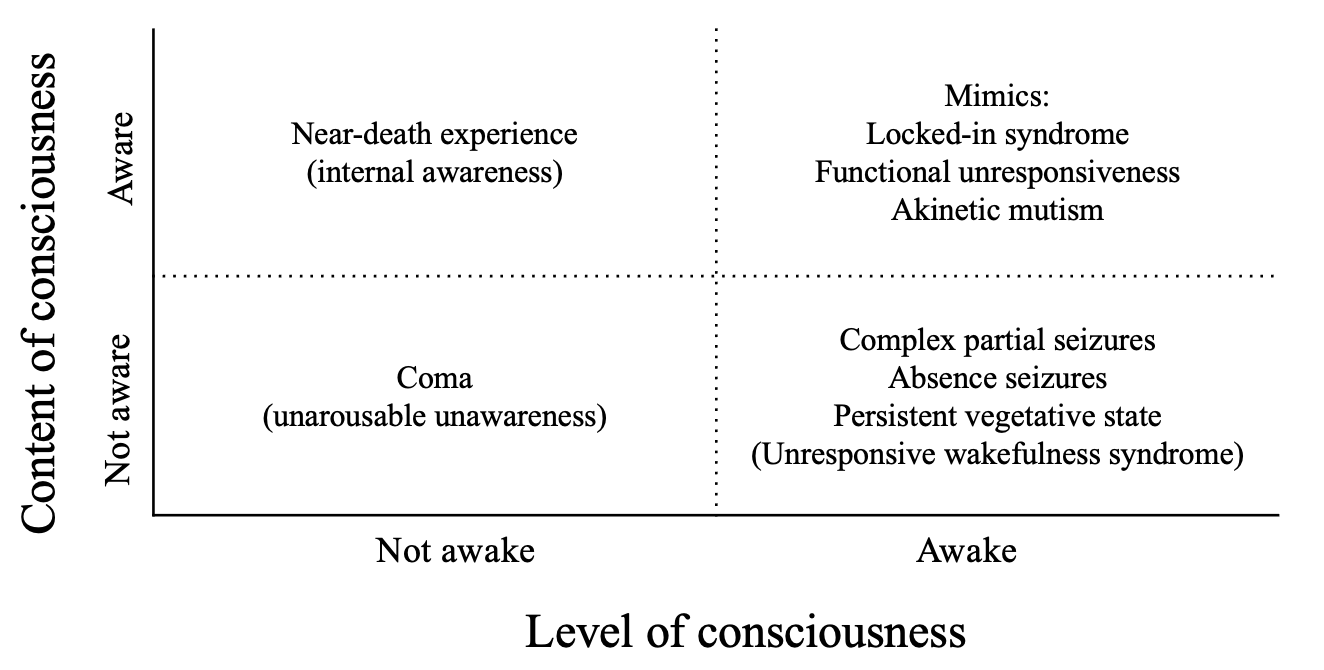


States exist in which level of consciousness and content of consciousness are either both impaired or both intact. Reduced level of consciousness with reduced content of consciousness typically describes *coma*, although some variation in the definition as to “unarousable *unresponsiveness*” [20] compared with “unarousable *unawareness*” exists [21]. Pathological states that may mimic disturbances of consciousness, which have intact level of consciousness and intact content of consciousness (below).

It is possible for one component of consciousness to be impaired, with relative preservation of the other component. Intact level of consciousness and reduced content of consciousness may occur in focal seizures with impaired awareness, absences seizures, somnambulation and a persistent vegetative state. In a minimally conscious state there is intact level of consciousness, with low, and potentially fluctuating, levels of awareness the quality of which is difficult to determine clinically [17]. Reduced level of consciousness and preserved content of consciousness is uncommon, but may be present in near-death experiences [1].

**Supplementary Information 6 –** Ethical considerations and future directions

Disorders of consciousness are associated with multiple ethical considerations [22]. Among these ethical issues, are situations pertaining to the demonstration of consciousness on medical imaging or neurophysiological testing (namely electroencephalogram) in the absence of clinical evidence of consciousness. This state may be referred to as covert awareness. However, the state has been referred to by multiple other names including *covert consciousness*, *cognitive motor dissociation*, *functional locked-in syndrome,* and *higher-order cortex motor dissociation* [23]. While this article focusses on clinical examination and communication, the existence of covert awareness highlights the importance of functional MRI and electroencephalogram in the evaluation of disorders of consciousness. This importance is reflected in current guideline statements [24]. Scoring systems, such as the AVCM scoring system [25], are now being developed that consider the presence or absence of findings such as cognitive content, agnostic to the method of evaluation (e.g., clinical examination, functional MRI, or EEG). However, this type of scoring system raises multiple further significant ethical considerations with respect to the equity of access to advanced neuroimaging and neurophysiological investigations [26].

**Supplementary Information References:**

1. Martial C, Cassol H, Laureys S, Gosseries O (2020) Near-Death Experience as a Probe to Explore (Disconnected) Consciousness. Trends Cogn Sci 24 (3):173-183. doi:10.1016/j.tics.2019.12.010

2. Cadena RS, Sarwal A (2017) Emergency Neurological Life Support: Approach to the Patient with Coma. Neurocrit Care 27 (Suppl 1):74-81. doi:10.1007/s12028-017-0452-1

3. Young G (2022) Stupor and coma in adults. UpToDate. 2022

4. Formica F, Pozzi M, Avantaggiato P, Molteni E, Arrigoni F, Giordano F, Clementi E, Strazzer S (2017) Disordered Consciousness or Disordered Wakefulness? The Importance of Prolonged Polysomnography for the Diagnosis, Drug Therapy, and Rehabilitation of an Unresponsive Patient With Brain Injury. J Clin Sleep Med 13 (12):1477-1481. doi:10.5664/jcsm.6854

5. Teasdale G, Jennet B (1974) Assessment of coma and impaired consciousness. A practical scale. Lancet 13 (2):81-84

6. American College of Surgeons CoT (1997) Advanced trauma life support manual, vol 6. American College of Surgeons, Chicago, IL

7. Kalmar K, Giacino JT (2005) The JFK Coma Recovery Scale--Revised. Neuropsychol Rehabil 15 (3-4):454-460. doi:10.1080/09602010443000425

8. Rappaport M, Dougherty A, Kleting D (1992) Evaluation of coma and vegetative states. Arch Phys Med Rehabil 73:628-634

9. Wijdicks EF, Bamlet WR, Maramattom BV, Manno EM, McClelland RL (2005) Validation of a new coma scale: The FOUR score. Ann Neurol 58 (4):585-593. doi:10.1002/ana.20611

10. Kelly CA, Upex A, Bateman DN (2004) Comparison of consciousness level assessment in the poisoned patient using the alert/verbal/painful/unresponsive scale and the Glasgow Coma Scale. Ann Emerg Med 44 (2):108-113. doi:10.1016/j.annemergmed.2004.03.028

11. Nuttall AG, Paton KM, Kemp AM (2018) To what extent are GCS and AVPU equivalent to each other when assessing the level of consciousness of children with head injury? A cross-sectional study of UK hospital admissions. BMJ Open 8 (11):e023216. doi:10.1136/bmjopen-2018-023216

12. Ramazani J, Hosseini M (2019) Comparison of full outline of unresponsiveness score and Glasgow Coma Scale in Medical Intensive Care Unit. Ann Card Anaesth 22 (2):143-148. doi:10.4103/aca.ACA_25_18

13. Gill MR, Reiley DG, Green SM (2004) Interrater reliability of Glasgow Coma Scale scores in the emergency department. Annals of Emergency Medicine 43 (2):215-223. doi:10.1016/s0196-0644(03)00814-x

14. Namiki J, Yamazaki M, Funabiki T, Hori S (2011) Inaccuracy and misjudged factors of Glasgow Coma Scale scores when assessed by inexperienced physicians. Clin Neurol Neurosurg 113 (5):393-398. doi:10.1016/j.clineuro.2011.01.001

15. Bledsoe BE, Casey MJ, Feldman J, Johnson L, Diel S, Forred W, Gorman C (2015) Glasgow Coma Scale Scoring is Often Inaccurate. Prehosp Disaster Med 30 (1):46-53. doi:10.1017/S1049023X14001289

16. Di Perri C, Thibaut A, Heine L, Soddu A, Demertzi A, Laureys S (2014) Measuring consciousness in coma and related states. World J Radiol 6 (8):589-597. doi:10.4329/wjr.v6.i8.589

17. Giacino JT, Ashwal S, Childs N, Cranford R, Jennett B, Katz DI, Kelly JP, Rosenberg JH, Whyte J, Zafonte RD, Zasler ND (2002) The minimally conscious state: Definition and diagnostic criteria. Neurology 58 (3):349-353

18. Mat B, Sanz LRD, Arzi A, Boly M, Laureys S, Gosseries O (2022) New Behavioral Signs of Consciousness in Patients with Severe Brain Injuries. Semin Neurol 40 (3):259-272

19. Kopelman M (2002) Disorders of memory. Brain 125 (10):2152-2190

20. Rabinstein AA (2018) Coma and Brain Death. Continuum 24 (6):1708-1731

21. Wijdicks EF (2010) The bare essentials: coma. Pract Neurol 10 (1):51-60. doi:10.1136/jnnp.2009.200097

22. Young MJ, Bodien YG, Giacino JT, Fins JJ, Truog RD, Hochberg LR, Edlow BL (2021) The neuroethics of disorders of consciousness: a brief history of evolving ideas. Brain 144 (11):3291-3310. doi:10.1093/brain/awab290

23. Schnakers C, Bauer C, Formisano R, Noe E, Llorens R, Lejeune N, Farisco M, Teixeira L, Morrissey AM, De Marco S, Veeramuthu V, Ilina K, Edlow BL, Gosseries O, Zandalasini M, De Bellis F, Thibaut A, Estraneo A (2022) What names for covert awareness? A systematic review. Front Hum Neurosci 16:971315. doi:10.3389/fnhum.2022.971315

24. Kondziella D, Bender A, Diserens K, van Erp W, Estraneo A, Formisano R, Laureys S, Naccache L, Ozturk S, Rohaut B, Sitt JD, Stender J, Tiainen M, Rossetti AO, Gosseries O, Chatelle C, Ean Panel on Coma DoC (2020) European Academy of Neurology guideline on the diagnosis of coma and other disorders of consciousness. Eur J Neurol 27 (5):741-756. doi:10.1111/ene.14151

25. Kondziella D, Menon DK, Helbok R, Naccache L, Othman MH, Rass V, Rohaut B, Diringer MN, Stevens RD, contributing collaborators of the Curing Coma C (2021) A Precision Medicine Framework for Classifying Patients with Disorders of Consciousness: Advanced Classification of Consciousness Endotypes (ACCESS). Neurocrit Care 35 (Suppl 1):27-36. doi:10.1007/s12028-021-01246-9

26. Farisco M, Salles A (2022) American and European Guidelines on Disorders of Consciousness: Ethical Challenges of Implementation. J Head Trauma Rehabil 37 (4):258-262. doi:10.1097/HTR.0000000000000776
